# The causal cognition of wrong doing: incest, intentionality, and morality

**DOI:** 10.3389/fpsyg.2015.00136

**Published:** 2015-02-18

**Authors:** Rita Astuti, Maurice Bloch

**Affiliations:** Department of Anthropology, London School of Economics and Political Science, London, UK

**Keywords:** intentionality, incest, morality, causal cognition, anthropology, Madagascar

## Abstract

The paper concerns the role of intentionality in reasoning about wrong doing. Anthropologists have claimed that, in certain non-Western societies, people ignore whether an act of wrong doing is committed intentionally or accidentally. To examine this proposition, we look at the case of Madagascar. We start by analyzing how Malagasy people respond to incest, and we find that in this case they do not seem to take intentionality into account: catastrophic consequences follow even if those who commit incest are not aware that they are related as kin; punishment befalls on innocent people; and the whole community is responsible for repairing the damage. However, by looking at how people reason about other types of wrong doing, we show that the role of intentionality is well understood, and that in fact this is so even in the case of incest. We therefore argue that, when people contemplate incest and its consequences, they simultaneously consider two quite different issues: the issue of intentionality and blame, and the much more troubling and dumbfounding issue of what society would be like if incest were to be permitted. This entails such a fundamental attack on kinship and on the very basis of society that issues of intentionality and blame become irrelevant. Using the insights we derive from this Malagasy case study, we re-examine the results of Haidt’s psychological experiment on moral dumbfoundedness, which uses a story about incest between siblings as one of its test scenarios. We suggest that the dumbfoundedness that was documented among North American students may be explained by the same kind of complexity that we found in Madagascar. In light of this, we discuss the methodological limitations of experimental protocols, which are unable to grasp multiple levels of response. We also note the limitations of anthropological methods and the benefits of closer cross-disciplinary collaboration.

## INTRODUCTION

This paper is about the role that intentionality plays in causal reasoning and, more particularly, in reasoning about, and responding to, acts of wrong doing. In the modern Western legal tradition and in Western folk thinking more generally, a sharp distinction is drawn between doing wrong intentionally and doing wrong through negligence or accident. For example, the English legal code considers murder and man-slaughter to be quite different and to merit an altogether different punishment. Although this distinction is often taken to be universal, some recent anthropological findings have challenged this assumption, showing that in some cultural contexts people only care about the effects of an action, not about the intentions behind it (or lack thereof; e.g., Danziger, 2006; Walker, 2015).

In this paper, we shall explore the proposition that considerations of intentionality are not a universal component of causal reasoning about wrong doing, by looking at an ethnographic case we are familiar with: the case of Madagascar. In the last part of the paper, we use our ethnographically specific conclusions on intentionality to reconsider some classic work in the social psychology of morality.

Before proceeding, a few clarifications about terminology and methodology are in order. By *intentionality* we mean “having the intention to act in a certain way or to cause a certain outcome,” as opposed to accidentally doing so. By *causal cognition* we mean the folk understandings of what causes certain events to take place. In the case of actions understood to be brought about by people, causal cognition concerns the understanding of the link between the actor and her acts. By *reasoning about wrong doing* we mean the assessment by members of a community of what caused the wrongdoing and what to do about it.

Regarding the methodology, what follows is based on our long-term ethnographic fieldwork in three different regions of Madagascar: Astuti, among the Vezo on the Western coast; Bloch, among the Merina and the Zafimaniry of the central highlands. Although there are significant differences between these populations with regards to their livelihoods, their relation to the state, their kinship system, and much more, in this paper we draw on ethnographic evidence that is equally valid across our field sites and, for ease of exposition, we refer to Madagascar and the Malagasy people in an undifferentiated manner (for the purpose of the present discussion, “people” refers to adults, men and women alike). Ethnographic fieldwork is based on the long-term engagement with communities of people who allow the anthropologist into their lives; the evidence it generates is gleaned through the gradual transformative process by which the anthropologist learns to move, speak, eat, sleep, dance, trade, fish, plant, tend animals, attend births and funerals, and so on, competently, as if she was a member of that community (for more details, see, e.g., Bloch, 1992; Astuti, 1995). This apprenticeship is aided by observation, participation, and by asking questions. With reference to the specific topic of this paper, we derive our conclusions from having learnt ourselves how to live morally in these communities, from having witnessed moral outrage or anxiety, from having asked explanations for decisions already made or for predictions of future behavior, and from having engaged people informally in counterfactual reasoning and other thought experiments in the course of our everyday interactions with them. This methodology yields in-depth and diffused knowledge, which cannot be quantified or statistically analyzed.

## INCEST

For reasons that will become clear, we start with the case of incest.

The definition of what counts as incest varies across Madagascar. In some parts of the island, the children of two brothers can marry, while this would be regarded as an incestuous union elsewhere. People are aware of these differences; but they are also aware that all Malagasy people, in so far as they are “people” and not “animals,” have a taboo against at least some sexual unions among kinspeople. Breaching this taboo causes terrible things to happen: crops fail, canoes overturn at sea, children die, women’s fertility dries up, infants are born with horns on their heads or humps on their backs.

Such catastrophic consequences unfold irrespective of whether the people who committed incest did so knowingly and intentionally. In cases of distant incest (for example, when the genealogical relation goes back three or four generations), the people involved may have no idea that they are related, hence that they are committing incest. In such cases, it is the misfortune that follows which reveals that, in fact, the incest taboo has been breached. Indeed, the severity of the breach is not calculated *a priori* by strict genealogical reckoning, but *post facto* by observing the extent of the harm that befalls the community and the significance of the atonement that is needed to put things right.

Similarly, in the course of informal conversations about incest, we told a couple of our Malagasy interlocutors a story about two siblings who get separated at birth and, later in life, meet and end up liking and having sex with each other. The judgment was that the two people are not at fault because they do not know that they are brother and sister; nonetheless, their act will cause terrible misfortune on their children and on their families. Notably, the fact that a large number of innocent people are expected to be affected by the wrongdoing of the culprits, underscores the conclusion that intentionality does not mediate between the cause (incest) and its effects (harm). Correspondingly, a large number of innocent people are responsible for undertaking the difficult (expensive, dangerous, stressful) ritual work that is required to repair the damage and put things right again.

All in all, it seems that when the Malagasy people we know reason about incest, predict and act upon its consequences, considerations of intentionality are simply beside the point. This is shown in three ways: first, harm follows irrespective of whether people are aware that they are breaching the incest taboo; second, harm befalls on people who have not themselves committed incest; third, the costs of ritual reparation befall on large numbers of innocent people. Incest, therefore, is prima facie a perfect endorsement of the claim that considerations of intentionality are not a universal component of causal reasoning about wrong doing.

## MUNDANE ACTS OF WRONG DOING

One might be tempted to conclude that Malagasy adults do not distinguish between intentional and accidental acts of wrong doing. This conclusion, however, is unsupported by evidence that comes from other contexts of social life, where the distinction between wrong doing that is committed intentionally and wrong doing that happens accidentally is clearly drawn and taken into account.

To tease out this distinction, we asked a few of our informants whether the punishment that follows an intentional act of mild aggression (kicking over somebody’s bucket of water) as opposed to an accidental one (stumbling over somebody’s bucket of water) is equivalent or different. In the discussions that were sparked by this hypothetical scenario, people reasoned that if the bucket gets broken, the person will have to replace it in both cases. But the process by which this happens will be very different: if it was an accident, the person will say sorry and will volunteer to replace the bucket, explaining that she did not see it; if it was an intentional act, a fight will ensue and the victim will take the perpetrator to the village assembly, where a more serious punishment might be dispensed (e.g., a monetary compensation in addition to the replaced bucket). In a different conversation, the following scenarios were presented: two neighbors own two identical chickens. In one scenario, one of them accidentally kills the chicken that belongs to the other person, while in the other scenario, the killing of the other person’s chicken happens knowingly and intentionally. When asked what would happen if the two cases were brought to the village assembly, our interlocutors said that in the case of the first (unintentional) killing, there would be no reason to go to the village assembly and that there would be no punishment either. The person who made the mistake would say sorry and give her chicken to the other and that would be the end of the story.

In such mundane cases, intentionality thus matters a great deal. This does not mean that the distinction between intentional and accidental wrongdoing can always be drawn with clarity: people can say that they did not see the bucket they stumbled over or that they did not know that the chicken they killed was not theirs, while in reality they saw the bucket and knew that the chicken was their neighbor’s. In other words, people can lie about their state of knowledge and about their intentions. But this uncertainty does not invalidate the distinction between intentional and accidental causality.

## ANCESTRAL TABOOS

Somewhere in between the case of incest—where intentionality seems to be beside the point—and the case of mundane acts of wrongdoing—where the distinction between intentional and accidental acts is paramount—there is the case of the breach of ancestral taboos.

Across Madagascar, people inherit a host of taboos from their ancestors, which determine which food they cannot eat, which animals they cannot kill, which words they cannot speak, which trees they cannot cut down, which color they cannot wear, and so on. As discussed at length elsewhere (Astuti, 2007a), adults are aware that the only, but fundamentally moral, reason people follow ancestral taboos is to show respect to their ancestors, who are the true source of their being. There is nothing inherently wrong in eating chicken or pork (whereas there is something inherently wrong in committing incest); what is wrong is to disobey one’s ancestors who have stipulated—for whatever reason—that chicken or pork is forbidden.

Against this background, what difference does it make whether people breach a taboo intentionally or accidentally? The evidence on this is ambiguous, and interestingly so. On the one hand, some taboos work in a mechanistic fashion that seems to by-pass the intentions of the taboo violator. Take the following example: there are ancestral taboos that proscribe certain behaviors at sea, especially in the pursuit of the highly prized sea-turtle. The consequence of breaching one of these taboos is the failure to catch a sea-turtle ever again. This punishment, however, does not befall on the individual who breached the taboo, but on the canoe that carried that individual to sea (whether or not he is the owner of the canoe). The fact that the punishment is dispatched to the canoe (an artifact which is said to “breathe,” as it gently and rhythmically responds to the pressure of the waves, but which is not attributed a mind/spirit) suggests that the intentions, the knowledge or the ignorance of the wrongdoer is simply irrelevant.

But this conclusion does not go unchallenged. Consider the fact that young children do not suffer any consequence if they breach an ancestral taboo. This is because, being still “unwise” and lacking any understanding (this is how adults describe them), they do not know what taboos are, why it is important to follow them, how disrespectful it is to disobey the ancestors, and so on. As a result, because of the immature state of their minds, it is as if their taboos did not yet exist. This does not mean that children are never victims of ancestral punishment; they are, but as a result of their parents’ and elders’ wrongdoing.

The same point—that breaching a taboo is of consequence only if it is done knowingly and intentionally—emerges from the result of the following study (described in Astuti, unpublished). Fifteen adult participants were told a story in which an infant is found abandoned in the forest and is raised by people who know nothing about his birth origins; they were then asked: will the taboos of this child’s birth parents (specifically, the taboo against eating pork or chicken) affect the child when he grows up or not? The overwhelming majority of adults (80%) responded that the child will not be affected by the taboo. They explained that the birth father’s taboo has been lost and that it will not work on the child because he does not know about it. Only the remaining handful of participants reasoned otherwise and suggested that when the child will eventually, if unknowingly, eat the food that was taboo for his birth father, he will become ill or crazy. The existence of the taboo will then “come out” and will be seen and explained by a diviner. In other words, the unintentional breach of a food taboo caused something like an allergic reaction—mechanically and irrespective of anyone’s intentions. But this was a minority view.

## ANOTHER LOOK AT INCEST

We have established that, when reasoning about acts of wrong doing and when considering what actions might follow (e.g., reparation, punishment, mediation), the Malagasy people we have worked with take into account whether such acts were undertaken intentionally or not. This finding forces us to ask why, then, intentionality does not seem to be taken into account in the case of incest. We will explore this question through two complementary moves: first ethnographically and then by way of a more theoretical reflection, which generates a testable hypothesis.

The word that Malagasy adults will almost certainly always use when discussing incest and contemplating its effects, is *loza*. The dictionary definition of this term is “calamity” or “disaster”; the verb for committing incest (*mandoza*) thus literally translates as causing a calamity or a disaster. This terminology expresses quite starkly the horror of incest: that incest causes everything to go wrong; that, in Hamlet’s words, when incest occurs “the time is out of joint” (see Wolf, 2014, p. 77 ff. for a range of ethnographic examples that express a similar sentiment).

As mentioned earlier, the consequences of incest are indeed understood as catastrophic: people’s livelihood, health, and reproduction are threatened. And yet, when asked why this is so, our Malagasy interlocutors are stumped—or dumbfounded, to use a term used in the psychological literature on moral reasoning (e.g., Haidt et al., unpublished; Haidt, 2001). In other words, they are unable to give a single and sufficient account of the relationship between cause (the breach of the taboo) and effect (*loza*). Instead, they come up with a multitude of answers that restate the necessity of the taboo and which do not satisfactorily explain (either for them or for us) the enormity of what incest brings about.

To try to understand the source of our informants’ dumbfoundedness, we turn to a theoretical discussion of the nature of human sociality. As argued elsewhere by Bloch (2008), among humans the social is fundamentally different from what it is among other primates. In the case of the latter, social roles are only perceived as existing in the here and now, and only so long as the individuals who fill them are capable of maintaining their position. Among humans, by contrast, social roles have a kind of imaginary existence that extends beyond the here and now: roles survive their incumbents; they extend beyond the life cycle, the frailty, the shortcomings of any one individual that inhabits them. In other words, they are experienced as having transcendental permanence. This, Bloch argues, is the result of the uniquely human capacity for imagination (see also Rakoczy, 2008; Wyman and Rakoczy, 2011, for a cognate point).

In the kind of Malagasy communities where we work, kinship and its roles—ancestor, elder, father, mother, mother’s brother, wife, husband, father- and mother in-law, son- and daughter-in-law, son, daughter, grandchild, and so on—are experienced as this form of transcendental sociality. Kinship is transcendental because it extends back in time and is projected into the future, thus seeming to involve a kind of unquestionable permanence beyond the biological lives of those who fill specific kinship roles at any one time; it is transcendental because its extension and temporal reach negate the experience of the fluidity of life in the here and now, even though such extension and temporal reach can only be experienced in the imagination and during circumscribed ritual acts that produce vivid snapshots of the transcendental order; and it is transcendental because, irrespective of who he might be as an individual (poor, weak, unsuccessful, mean-spirited) a father-in-law is a father-in-law, who deserves respect and deference from his son-in-law (who might be wealthier, stronger, more successful and deeply resentful).

Ordinary life in Malagasy villages is not experienced only through this transcendental sociality. The now-on-now-off temporality of human life is fully recognized: how could it not be when people see babies turn into adults and adults turn into lifeless bodies; people’s non-transcendental personalities matter: they please and annoy in the same measure; the fickleness of kinship relations is an ever ending topic of conversation: despite being a kin, she did not behave in kin-like fashion. And yet, even though kinship in its transcendental form appears to negate one’s daily experience, it also appears to be essential to people’s very existence—“if people are people,” as our Malagasy informants would put it, they have to have a permanent system of kinship that extends through time and that slots people into roles that have permanence and fixity. By transcending the fluid, largely unpredictable interactions that make up everyday life, transcendental kinship provides an image, however vague, of a stable and lasting order and seems to afford certainty about what people ought to do and how they should behave—as mothers and fathers, as children and grandchildren.

This is why incest leaves people dumbfounded: it is because incest is felt to attack the foundational principles of kinship and, by attacking kinship at its foundation, it is felt to threaten the transcendental in its entirety. In ethnographic terms, as we have seen, incest is said to cause *loza*: calamity and disaster. In more abstract and theoretical terms, we now propose, incest is perceived as a threat to the very fabric of human sociality. This is because the possibility of incest evokes a world where everything and anything is allowed; a world where there are no rules, no respect for elders or for ancestors, who are the source of one’s own existence. Note the difference between the breach of the incest taboo and the breach of ancestral taboos: as we noted above, ancestral taboos are the result of decisions made by one’s ancestors (e.g., that we should not eat pork). There is nothing dumbfounding about the prospect of breaching one of these taboos, because doing so amounts to a single act of disobedience (indeed, if one manages to get away with it, such disobedience can be experienced as enjoyable and liberating). What would be dumbfounding for our informants is the prospect of breaching all ancestral taboos in an act of collective defiance, thus defying the fundamental principle of age hierarchy. As with incest, such a scenario would amount to a wholesale attack on kinship, which would cause generalized *loza* and would question the very humanity of those concerned.

Returning to incest: at issue is not so much who can and cannot have sex with whom, or how incest should be punished; at issue is the much more fundamental question of whether any rule at all is legitimate. The very fact that incest can occur seems to invite the thought that the rules we live by may be just flimsy fictions; that, perhaps, the incest taboo and the marriage rules that ensure its avoidance are just a convention. Indeed, the possibility of such a challenge seems to be implicit in the recognition that people in different parts of Madagascar have different definitions of what counts as incest. This line of reasoning is dumbfounding because, if one starts to ask these kinds of questions, social life begins to unravel and nothing is safe.^1^

From this perspective—that of the possibility of incest as a total attack on the social—we can understand (and could have predicted) all the three ways we mentioned earlier in which intentionality is bypassed: it makes sense that, if incest occurs, harm will follow irrespective of whether it was committed intentionally or not; it makes sense that the catastrophic consequences of incest will affect everyone; and it makes sense that everyone is responsible for trying to put the world back together again. From this perspective, we can expect intentionality to become irrelevant because the breach is too enormous, the consequences too shattering, the repair work too essential.^2^

## ANOTHER LOOK AT INTENTIONALITY

We have argued that, although our Malagasy informants take intentionality into account when considering acts of wrong doing, its relevance seems to fade away when incest is concerned, because of incest’s cosmic consequences.

We now need to qualify our argument, by recognizing that intentionality can play a role in people’s reasoning about incest. Across Madagascar, it is the elders’ responsibility to make young people aware of the individuals they are in a taboo relationship with. As soon as children reach the age when they are deemed to be interested in sex, they will be told: those people, they are taboo to you. What, then, if these young people intentionally disregard what they have been told and start up an incestuous relationship? When people envisage this possibility, they pass strong judgment on the irresponsibility of those youngsters who knowingly disregard the warnings of their elders to indulge the attraction they feel for one another. Somewhat predictably, today’s youths are deemed to be more selfish and immoral than those of the past; they are accused of breaching basic taboos that would ensure that brothers and sisters (anyone who is referred by these terms in the expansive web of classificatory kinship) do not come into any kind of sexually inflected association with one another. For example, it is bad enough that girls nowadays wear trousers, which expose their groin to their male kin, but it is shocking that a brother and a sister should share the very same garment. When people discuss such cases, they express a sense of outrage, along the lines: “what do they think they are doing, behaving like that?” As people express these worries, they focus on the deliberate, intentional disregard for the rules that are meant to protect people, young and old.

Another common trope is that, if a couple is found to have committed incest, whether knowingly or unknowingly, they will be asked to immediately separate and bring their relationship to an end. But what do youngsters do nowadays? They will retort that, if forced to separate, they will commit suicide. Their stubbornness is deemed unreasonable and particularly wicked, because they selfishly and intentionally force on their families an impossible choice: to cause their children to take their own life or to condone their incestuous relationship.

Whether wrong doing is done intentionally or not can thus be taken into account even in the case of incest. But the point is that attributing blame is a quite different concern than imagining a world without any incest rule, where what is experienced as necessary for one’s collective existence as human beings is under threat. Attributing blame, in other words, is a quite different matter than dealing with *loza*.

## CAUSAL COGNITION AND INTENTIONALITY

Through the analysis of our data from Madagascar, we have made the following arguments: that the way people reason about, and respond to, incest is, prima facie, an example of causal reasoning being decoupled from intentionality; nonetheless, this does not warrant the conclusion that Malagasy people have a radically different form of causal cognition that is blind to intentionality; evidence that intentionality is taken into account comes from the way people handle mundane forms of wrong doing as well as the breach of a host of ancestral taboos; indeed, we have also shown that considerations of intentionality are present in the way people assess culpability even in the case of incest.

What the discussion above shows is that, when talking about, and taking actions in response to incest, our informants may be thinking about two quite different kinds of thing: they may be deliberating about who did what and who should be blamed, and they may be evoking the catastrophic image of a world where incest is permitted. In the first instance intentionality is relevant, whereas in the second instance it is not. Our hypothesis—which can be tested cross-culturally—is that both of these responses are going to be present whenever people respond to incest: on the one hand, they will engage their everyday causal reasoning, while on the other hand they will be dumbfounded by the attack on the transcendental that incest instantiates.

Anthropologists, who study talk and action within lived contexts, are in a position, if they are so minded, to distinguish between these two responses. Because, at the back of their minds, they have a myriad of practices and discourses from their long term experience of sharing the life of the people they study, they can recognize when people switch, from instant to instant, from one type of discourse to the other. They might be able to distinguish between the two even when people, as they often do, draw on the two simultaneously. This is what we have shown in this paper, demonstrating the kind of understanding that anthropologists are positioned to contribute, as they observe and participate in the contexts where people reflect, talk and act jointly with others.

This kind of understanding, by contrast, is not easily generated by the methods typically used by psychologists. Such methods proceed by deliberately isolating subjects in controlled experimental settings, placing them outside any actual lived social context. Without the wider social context in which their experimental subjects think and act, psychologists are at risk of not actually understanding what their subjects say and do in the conduct of the experiment. In the next and final section, we illustrate this point with reference to a most famous case of moral dumbfoundedness.

## MORAL DUMFOUNDEDNESS RE-EXAMINED

In an influential paper, Haidt and two of his co-researchers (Haidt et al., unpublished manuscript) reported the results of an experiment with undergraduates from the University of Virginia, which became the cornerstone of Haidt’s (2001) “social intuitionist model of moral judgment.”

Briefly, students were told three different stories that called for a moral judgment (on whether the action depicted in the story was wrong) and they were presented with two situations that called for an action (which they could accept or refuse to undertake). One of the stories was the so called Heinz dilemma, which pitted the wrongness of theft against the necessity to save the life of one’s loved one. The expectation was that, in this case, participants would engage in dispassionate moral reasoning, evaluating the pros and cons of the two possible courses of action. By contrast, in the case of the other two stories—one about incest between brother and sister and the other about cannibalism—and in the case of the two actions—drinking from a glass of juice after a perfectly sterilized cockroach was dipped into it and signing off to the experimenter one’s soul after one’s death—the expectation was that participants would have a strong moral intuition that the action was wrong (which they did) and a strong rejection of the proposed actions (which they had), but that they would be unable to explain why. In other words, the prediction was that they would be morally dumbfounded.

The stories about incest and cannibalism were written to pre-empt and counteract the usual objections to such acts. For example, the story about incest said that the siblings took absolutely reliable precautions against the possibility of pregnancy; that they had sex in secret and that they never mentioned it to anyone else; that after the act, which they enjoyed, they decide not to do it again and that they went on to live very happy lives, feeling even closer to one another. Having judged that it was wrong for the brother and sister to have sex, the students proved unable to explain why this was so. They offered all the predicted standard arguments: the fact that the brother and sister might give birth to a deformed child; the fact that their act might offend the sensibilities of other people; the fact that the act would be detrimental to their long term relationship and their psychological well-being. Of course, all of these reasons had been ruled out by the story, and the experimenter, playing his scripted role as “devil’s advocate,” told them so. And yet, the students came back, again and again, trying to find new arguments, exploring what they soon recognized were “dead ends,” admitting that they did not know, that they could not explain (i.e., that they were dumbfounded), all the while growing increasingly frustrated, as evidenced by their facial expressions, their fidgeting behavior, nervous laughter and the like.

The interpretation of the students’ dumbfoundedness (which was also in evidence in the case of cannibalism and in the response to the two proposed actions, but which was absent in the case of the Heinz dilemma) was that, in responding to these specific scenarios, the students were guided by their emotions, their “gut feeling” that certain behaviors and actions were just wrong. Having made an intuitive, emotional judgment, they later searched, unsuccessfully, for some rational justification. Following Hume’s non-rationalist tradition, Haidt’s “social intuitionist model” thus posits that “moral judgment is caused by quick moral intuitions and is followed (when needed) by slow, ex post facto moral reasoning” (Haidt, 2001, p. 817).

We want to propose an alternative to this conclusion and to Haidt’s explanation of dumbfoundedness—an unquestionably real phenomenon—on the basis of our experience as anthropologists. Often enough, in the course of fieldwork, we witness our informants’ dumbfoundedness: they are unable to produce answers to our questions concerning why something is right or wrong, why they do what they do, or why they believe what they assert. It is thus very easy for us to imagine that our Malagasy informants, faced by the experimental situation that Haidt presented to his American subjects, would behave in very similar fashion. Taking the example of incest: they too would maintain that incest is very wrong, and they would continue to do so even if all the specific reasons they might come up with to explain why have been discounted one by one. They too might start fidgeting and grow frustrated, and might politely tell us that they do not know why, but that they know that it is so. However, in light of our ethnographic evidence and the analysis we have developed in this paper, we are wary of attributing their dumbfoundedness to the role of their emotional intuitions.

One thing we have learnt as anthropologists is that the first and most important step in any investigation is to interrogate whether the questions one asks are hitting the point, namely whether they address the issue one is investigating in a way that genuinely touches on the concerns of one’s interlocutors—even when, apparently, everyone is using the very same words. For example, one might want to question what it would actually mean to ask our Malagasy informants whether it is wrong for Julie and Mark—the sister and brother of Haidt’s experimental story—to have sex. Asked in this way, the question is about two individuals making a decision and acting in isolation. But while we are busy asking about Julie and Mark and recording the answers and the scrambling for some kind of justification, our informants might be thinking about something entirely different. They might be contemplating more profound and much more dumbfounding questions, lurking behind the question about Julie and Mark. The questions would be: what kind of society would this be where brothers and sisters can have sex? A society, a kinship and a family system where incest is acceptable? How could one live in such a place? The way our Malagasy informants would apprehend and respond to these questions is through the readily available concept of *loza* which, as we have argued above, evokes a state of complete social catastrophe caused by an outright attack on the transcendental. In other words, we are proposing that in responding to the Julia and Mark incest scenario, our Malagasy informants would be shifting away from a focus on two isolated individuals and the emotions triggered by their action, to a consideration of what a good society must be like. Their focus (including their emotional reaction), would be on people’s need for the apparent imaginary permanence of kinship, for the non-negotiable rules that protect it. In other words, their overriding concern would be to restate and reassure themselves that “for people to be people” society has to be grounded in the transcendental.

We would like to suggest that the situation is not entirely different for the students tested by Haidt and his colleagues. In the experiment, the students were put in a situation in which they had to decide, in complete isolation and away from any meaningful social context, whether Julie and Mark’s action was right or wrong. The reason they grew increasingly frustrated, we suggest, is that they were forced to pretend that the moral rules and concomitant emotions by which their social world is created and lived by, are generated by the students themselves, individually and on the grounds of having a good argument to back them up.

Some might argue that bringing our ethnographic experience of working in “holistic” communities in Madagascar to bear on the interpretation of experimental results obtained in “individualistic” university campuses in North America is preposterous. We do not think so. Even if there is no doubt that the students in Haidt’s experiment have grown up in a society where individualism is rhetorically hegemonic, it is nonetheless the case that they too must experience the social and its rules as originating not in their personal deliberations or private emotions, but in something that they can only grasp in the imagination. Provoked by the experiment’s “devil’s advocate” into finding logical reasons for their judgment, the students just gave up, since they know, or perhaps feel, that their individual and isolated opinion is really beside the point. Their dumbfoundedness signals that what they are thinking and care about is the need to align themselves, jointly with others, with what, ultimately and fundamentally, makes people people, namely, the transcendental.

## CONCLUSION

In this paper, we have made a strong case for the value of the anthropological approach, showing what insights it can offer to psychologists. By way of conclusion, we want to acknowledge that the psychological approach has an important contribution to make to the work of anthropologists—the two should be brought into a fruitful dialectic relationship with each other. Specifically, anthropologists are easily tempted to use isolated bits of ethnographic evidence to reach doubtful psychological generalizations about the cognitive characteristics of the people they study. The use of psychological techniques and the awareness of psychological findings can provide a useful corrective to the ease with which anthropologists reach conclusions about radical cognitive differences. Here as in our previous work (e.g., Astuti, 2007b; 2009; Bloch, 2007, 2011; Astuti and Bloch, 2012), we hope to have demonstrated the fruitfulness of combining concerns and insights from both disciplines.

### Conflict of Interest Statement

The authors declare that the research was conducted in the absence of any commercial or financial relationships that could be construed as a potential conflict of interest.

## Footnotes

1 There is an obvious similarity between our analysis and Lévi-Strauss’s argument about incest—that the incest prohibition is the foundation of human society, which marked the transition from nature to culture. We need not follow him in the evolutionary dimension of his argument. But we note that in societies where kinship provides the overwhelming experience of the transcendental, people do feel that if incest were allowed to occur, then human society would collapse. Life would not be human life any longer.

2 Note that our analysis does not exclude the possibility that humans have evolved a natural aversion toward incest: see Wolf (2014) for a recent elaboration of this argument; in and of itself, such a natural aversion does not explain what we are trying to explain, that is, why the intentionality of the wrong-doer is not relevant when reasoning about incest.
